# Factors affecting costs and utilization of type 2 diabetes healthcare: a cross-sectional survey among 15 hospitals in urban China

**DOI:** 10.1186/1472-6963-10-244

**Published:** 2010-08-20

**Authors:** Weibing Wang, Chaowei Fu, Haijing Zhuo, Jianfeng Luo, Biao Xu

**Affiliations:** 1 School of Public Health, Fudan University, China; 2 Cardiovascular Research Institute, University of California San Francisco, USA

## Abstract

**Background:**

Type 2 Diabetes mellitus (T2DM) affects persons of all ages, while also placing heavy economic burdens on national economies and healthcare systems. The study aims to investigate the determinants of direct medical cost (DMC), out-of-pocket (OOP) proportion of the cost, and healthcare utilization associated with T2DM.

**Methods:**

This cross-sectional study was conducted in four major cities in China. Eligible subjects were adult outpatients who received treatment at one of 15 sampled secondary or tertiary hospitals and consecutively enrolled between March 2007 and May 2007. Generalized estimating equations were used to determine impact factors associated with DMC and healthcare utilization.

**Results:**

Insurance schemes and receiving insulin therapy were significantly associated with a higher annual DMC of T2DM. For each increase in number of complications, there was about 33% increase in annual DMC. Insurance schemes were significantly associated with the proportions of DMC from pocket. A 7% significantly lower proportion of DMC was paid and 23% more clinic visits (AOR = 1.232, P < 0.001) were made by patients admitted at secondary hospitals than tertiary hospitals. The group with higher income (> 2000 CNY/month) paid 23% less from their pocket, compared with the lower income group. The number of complications also significantly increased the outpatient visits (AOR = 1.064, P < 0.001).

**Conclusions:**

It implies that preventing complications through the use of more effective treatment regimens is important in order to control the healthcare expenditures of the diseases. Healthcare reform needs to be focused on the medical insurance system and redistribution of patients in hospitals of different levels.

## Background

Type 2 Diabetes mellitus (T2DM) affects people at all ages. Nearly, 200 million people (or over 5% of the global adult population) have diabetes worldwide, and this number will increase up to 333 million (or 6.3% of the global adult population) by 2025 [1]. In the developing world, the prevalence was highest in Europe and Central Asia and lowest in Sub-Saharan Africa. In China, the total number of diabetes patients is currently 92.4 million [2,3] and expected to double by 2030 [4].

T2DM is also a heavy economic burden on national economies and healthcare systems, especially in developing countries[4]. The direct healthcare costs of diabetes range from 2.5 to 15.0 percent of annual healthcare budgets, depending on local prevalence and sophistication of the treatments available[5]. In China, it is expected that the economic burden associated with T2DM will increase dramatically in the next few decades with the sharp increase of the number of diabetic patients. Previous studies suggested that healthcare expenses in people with diabetes were more than two times greater than that in those without [6,7]. Major costs for the disease include medical expenditures for blood glucose level control and additional medicine and service for diabetes related chronic complications, which may lead to excess costs and additional consumption of healthcare services [8].

Several factors were reported to have impact on overall economic burden in diabetic patients. Studies showed that level of Hemoglobin A1c (HbA1c) and insulin treatment, were highly associated with total medical costs [9-12]. Some other studies suggested sharing of health expenditure, an index of equity in healthcare financing, was associated with medical costs, and affected by clinical characteristics and demographic factors as well [13,14]. Unreasonable healthcare utilization, i.e., occurrence of hospitalization and number of outpatient visits, which was associated with the increased healthcare costs of T2DM [13,15], was affected by insurance schemes and income levels [16,17].

The burden of diabetes has been a big challenge on clinical and public health. So far, only limited studies have been reported to identify the impact factors of medical costs and healthcare utilization in T2DM population. Moreover, those studies rarely addressed the issues in the setting of developing countries. An analysis of the demographic, socioeconomic and clinical influencing factors that affect medical cost, sharing of expenditure and health utilizations will help better understand the increasing medical cost of T2DM, especially in developing countries. The purpose of our study is to investigate the determinants of direct medical cost (DMC), out-of-pocket proportion (OOP) of the cost, and healthcare utilization associated with T2DM in a population of T2DM outpatients by a cross-sectional survey conducted in four Chinese cities in order to help healthcare policy makers to control the high medical costs of T2DM and reduce the inequity across different socioeconomic populations.

## Methods

### Study Setting

This cross-sectional study was conducted in four major cities - Shanghai, Beijing, Guangzhou and Chengdu - located in eastern, northern, southern and western China, respectively.

### Study subjects

Eligible subjects were adult outpatients who received treatment at one of 15 secondary or tertiary hospitals and met WHO criteria for diagnosis of T2DM [18]. Patients consecutively enrolled from each hospital's outpatient clinics during 2 months period (March 2007 to May 2007).

### Subjects recruitment and data collection

Study coordinators with background of clinical medicine or preventive medicine were hired from each study site and trained by the investigators. Written informed consents were obtained from all patients before enrollment. Patients were interviewed face-to-face in the hospitals at admission using a survey designed by the School of Public Health, Fudan University. The survey included questions for patients' demographics, diabetes characteristics, existing complications caused by T2DM, and treatment history, as well as self-estimations of the cost of T2DM and its complications. This study was approved by the Ethics Committee of the School of Public Health at Fudan University.

### Outcomes

The primary outcome of the study was DMC, defined as the total expenditures for treating T2DM and related complications, including co-payment, diagnosis, treatment, diagnostic testing, prescription drugs and medical supplies. Secondary outcomes included OOP proportion and outpatient visits in recent 6 months. OOP proportion was measured as a nonreimbursable expense paid by a patient. It was calculated by a total of itemized DMC paid by the patients without reimbursement vs. total DMC in the past year. Outpatient visits in recent 6 months were measured by counting the times of visiting outpatient department because of T2DM or diabetes related complications during the past six months before the survey.

### Measurement

Demographic and socioeconomc characteristics, including marital status (e.g., "Currently, your marital status is...?"), education level (e.g., "Your level of education is...?"), employment (e.g., "Are you currently working?"), and monthly income (e.g., "Over the past 12 months, approximately how much was your personal income per month?") were examined to explore the factors for outcomes. Clinical characteristics (e.g., "During the past 12 months, did you take insulin therapy?"; "During the past 12 months, have you had the below concurrent disease(s) diagnosed by a doctor?"; "When had your first T2DM diagnosis been made?") was also included in the analysis.

China's healthy care institutions are divided into three levels (or tiers) in urban areas - primary institutions (community hospitals or health centres that provide basic healthcare services for communities), secondary institutions (regional hospitals that provide comprehensive medical care, medical education, and medical research for the regions), and tertiary institutions (cross-regional, providing comprehensive and specialized medical care with a high level of medical education and research functions) (Guo ZH 1990). The level of the hospitals where a patient enrolled to the study was also included in the analysis.

In addition, all subjects were asked to take a HbA1c test free of charge upon enrollment to the study. A finger tip blood sample (50ul) was collected from each patient and sent to an assigned center in each city for HbA1c test within 7 days using a standardized method with Bio-Rad Variant II. Re-test of HbA1c were regulated in the 301 Hospital in Beijing. Of all the subjects, 2% were randomly sampled for providing two finger blood samples at the same time, one for testing on-site, and the other for re-test. The intra-class correlation coefficient for the quality control (two tests) was 0.76, showing a good accordancy.

### Statistical analysis

All data were entered into a Chinese database (EpiData version 3.1) and transferred into STATA 9.0 statistical package (STATA Corp, College Station, TX, USA) for statistical analyses. Odds Ratios (OR) and their 95% confidence intervals (95% CI) were computed using the parameters' estimates of the generalized estimating equation (GEE) models and used for the interpretation of the results. A 2-sided p-value < .05 was considered statistically significant.

In this study, DMC and OOP proportion were measured as continuous variables that are uncensored, positive-valued, and skewed, while outpatient visits represent the number of independent events that occur during a fixed period of time (Table 1). Therefore, we used GEE models to determine impact factors associated with the direct medical cost and OOP proportion, with a Gamma distribution and log link function; a GEE model with Poisson distribution and loglinear function was performed to explore the factors affecting outpatient visits in recent 6 months. Considering the characteristics of each city, we managed city as a cluster variable in the GEE models.

**Table 1:** Distributions of the outcome variables

Variables	Mean(SD,)	Median(Quartiles)	Range	Skewness
DMC (CNY)	7926(13785)	4564(1916-9000)	219000	7.40
OOP proportions	0.47(0.39)	0.31(0.11-1.00)	1.00	0.33
Outpatient visits in recent 6 months	7.42(7.24)	6.00(3.00-10.00)	72	2.77

DMC: Direct medical costs, OOP: out-of-pocket

## Results

### General characteristics and mean annual DMC of the study participants

A total of 1530 subjects identified as type 2 diabetes patients were recruited in the study period. Among them, 1478 patients met the inclusion criteria and offered information on cost in analysis, of whom 41.7% were male and 58.7% enrolled in tertiary hospitals. Of the patients enrolled, 91.1% were taking medications and 32.9% were receving insulin therapy. Univariate analysis shows, patients receving insulin therapy, having one or more complications, suffering a long disease course (> 5 years) had a significantly higher annual DMC (Table 2). The higher income group (> 2000 CNY/month) had a higher coverage (88.0%) of urban employee insurance than the lower income group (77.9%).

**Table 2:** General characteristics and DMCs (CNY)of the subjects

Variables	Value	Number (%)	Mean annual DMC (SE)	P value*
City	Beijing	375 (24.6)	8413 (430)	0.142
	Guangzhou	376 (24.7)	8716 (829)	
	Shanghai	373 (24.5)	8095 (457)	
	Chengdu	400 (26.2)	6562 (975)	
Sex	Male	616 (41.7)	8410 (562)	0.254
	Female	862 (58.3)	7581 (465)	
Lifestyle intervention	Yes	854 (57.9)	7772 (446)	0.618
	No	622 (42.1)	8135 (592)	
Drug therapy	Yes	1346 (91.1)	7593 (357)	0.003^‡^
	No	130 (8.9)	11359 (1687)	
Insulin therapy	Yes	485 (32.9)	11047 (707)	< 0.001^‡^
	No	990 (67.1)	6389 (399)	
Medical insurance	Urban employee insurance	1261 (85.3)	8275 (394)	0.080
	Commercial insurance	18 (1.2)	8061 (1678)	
	Cooperative Medical Scheme	23 (1.6)	2965 (749)	
	None	176 (11.9)	6167 (1128)	
Individual monthly income (CNY)	< = 2000	1009 (68.3)	7523 (436)	0.099
	> 2000	469 (31.7)	8795 (629)	
Education level	< = 9 years	637 (43.1)	7805 (552)	0.758
	< 9 years	840 (56.9)	8028 (472)	
Employed	Yes	241 (15.9)	6571 (686)	0.093
	No	1234 (84.1)	8201 (408)	
Admission hospital	Secondary	196 (13.3)	9202 (669)	0.233
	Tertiary	1282 (86.7)	7759 (400)	
Complication(s)	Yes	792 (52.0)	10320 (633)	< 0.001^‡^
	No	732 (48.0)	5372 (276)	
Disease course	< = 5 years	580 (38.1)	6416 (528)	< 0.001^‡^
	> 5 years	944 (61.9)	8869 (478)	

Note: total number amounts may vary due to missing values; SE: Std. Err; *: P values were obtained from one-way ANOVA test.; ‡: P < 0.01.

### Multivariate analysis of factors associated with direct medical cost

Table 3 showed the influencing factors associated with annual DMC. After ajustment for clustering on city, those who were covered by commercial insurance (P < 0.001, vs. none of insurance) and received insulin therapy (P < 0.001, vs. without insulin therapy) were significantly associated with a higher annual DMC of T2DM, with 28% and 58% increase respectively. A significantlhy higher annual DMC was paid by male patients than female patients (P = 0.039). The employed outpatients paid 12% less (P = 0.047) annual DMC than unemployed subjects. Moreover, for each increase in number of complications (P < 0.001), there was a 33% increase in annual DMC.

**Table 3:** Factors associated with annual DMC estimated by GEE

Variable	AOR	SE	Z	P value	95% CI	
Insurance (Reference: none)						
Urban employee insurance or resident insurance	1.243	0.219	1.23	0.217	0.880	1.757
Commercial insurance*	1.277	0.053	5.86	< 0.001^‡^	1.177	1.386
Cooperative Medical Scheme	0.463	0.192	-1.85	0.064	0.205	1.046
Lifestyle intervention: yes/no	0.988	0.110	-0.11	0.915	0.795	1.229
Drug therapy: yes/no	1.029	0.046	0.64	0.524	0.942	1.124
Insulin therapy: yes/no	1.575	0.162	4.42	< 0.001^‡^	1.288	1.927
Hospital: secondary/tertiary	1.215	0.138	1.72	0.085	0.973	1.517
Gender: male/female	1.146	0.076	2.06	0.039^†^	1.007	1.305
Employed/non-employed	0.880	0.057	-1.99	0.047^†^	0.775	0.998
Individual income: higher/lower	0.964	0.080	-0.44	0.657	0.820	1.134
Age (years)	1.002	0.004	0.52	0.600	0.994	1.011
Disease course (years)	0.996	0.008	-0.47	0.637	0.982	1.011
Education	1.017	0.093	0.19	0.849	0.851	1.217
Complication number	1.325	0.084	4.42	< 0.001^‡^	1.169	1.501
HbA1c value	0.980	0.039	-0.51	0.612	0.906	1.060

Note: Estimated by gamma log link function, using generalized estimating equations; adjusted for clustering on city.

DMC: direct medical costs; AOR: Adjusted Odds ratio; 95% CI: 95% confidence interval of AOR; SE: Std. Err; OOP: out-of-pocket; †: P < 0.05; ‡: P < 0.01. *: When patients simultaneously had commercial insurance and other plans, they were categorized into the other groups.

### Multivariate analysis of factors affecting OOP proportion of direct medical cost

The AOR of factors asscociated with OOP proportion from DMC and its 95% CI were listed in Table 4. After adjustment for clustering on city, a 7% significantly lower proportion of DMC was paid by patients admitted at secondary hosptals than tertiary hospitals (P < 0.001). A significantly 8% higher proportion of DMC was paid by employed patients than unemployed patients. The group with higher income (> 2000 CNY/month) paid 26% less from their pocket, compared with the group with lower income. Moreover, for each increased year in disease course, we observed about 1% decrease in the OOP proportion out of DMC.

**Table 4:** Factors affecting OOP of annual DMC estimated by GEE

Variable	AOR	SE	Z	P value	95% CI	
Insurance (Reference: none)						
Urban employee insurance or resident insurance	0.556	0.113	-2.89	0.004^‡^	0.374	0.828
Commercial insurance*	0.616	0.131	-2.28	0.023^†^	0.406	0.935
Cooperative medical scheme	0.812	0.071	-2.38	0.017^†^	0.684	0.964
Lifestyle intervention: yes/no	0.992	0.096	-0.08	0.937	0.821	1.199
Drug therapy: yes/no	0.966	0.119	-0.28	0.780	0.760	1.229
Insulin therapy: yes/no	0.994	0.039	-0.16	0.876	0.920	1.074
Hospital: secondary/tertiary	0.934	0.009	-7.26	< 0.001^‡^	0.917	0.951
Gender: male/female	0.950	0.041	-1.20	0.229	0.873	1.033
Employment: employed/unemployed	1.083	0.040	2.14	0.033^†^	1.007	1.165
Individual income: higher/lower	0.735	0.099	-2.29	0.022^†^	0.565	0.956
Age (years)	0.988	0.004	-3.36	0.001^‡^	0.981	0.995
Disease course (years)	0.991	0.004	-2.40	0.016^†^	0.984	0.998
Education	0.967	0.028	-1.19	0.235	0.914	1.022
Complication number	1.026	0.019	1.39	0.166	0.989	1.064
HbA1c value	1.019	0.018	1.05	0.294	0.984	1.055

Note: Estimated by gamma log link function, using generalized estimating equations; adjusted for clustering on city.

AOR: Adjusted Odds ratio; 95% CI: 95% confidence interval of AOR; SE: Std. Err; OOP: out-of-pocket; †: P < 0.05; ‡: P < 0.01. *: When patients simultaneously had commercial insurance and other plans, they were categorized into the other groups.

### Multivariate analysis of factors associated with outpatient visits in recent 6 months

The factors associated with times of outpatient visits were given in Table 5. After adjustment for clustering on city, outpatients admitted at secondary hospitals had 23% more clinic visits (AOR = 1.232, P < 0.001) than those at tertiary hospitals. The number of complications also significantly increased the outpatient visits (AOR = 1.064, P < 0.001). There was a 6% increase of hospital visits for each number increase of complications in outpatients.

**Table 5:** Factors associated with the outpatient visits in recent 6 months estimated by GEE

Variable	AOR	SE	Z	P value	95% CI	
Insurance						
Urban employee insurance or resident insurance/none	1.182	0.197	1.01	0.314	0.853	1.639
Commercial insurance/none*	1.014	0.148	0.10	0.924	0.762	1.349
Cooperative Medical Scheme/none	0.996	0.293	-0.01	0.988	0.559	1.774
Lifestyle intervention: yes/no	1.076	0.075	1.06	0.290	0.939	1.234
Drug therapy: yes/no	0.961	0.074	-0.52	0.603	0.827	1.117
Insulin therapy: yes/no	1.136	0.135	1.07	0.285	0.899	1.434
Hospital: secondary/tertiary	1.232	0.036	7.10	< 0.001^‡^	1.163	1.306
Gender: male/female	0.971	0.049	-0.59	0.553	0.879	1.071
Employed/non-employed	0.926	0.076	-0.94	0.349	0.788	1.088
Individual income: higher/lower	1.170	0.172	1.07	0.285	0.877	1.562
Age (years)	1.005	0.004	1.26	0.209	0.997	1.012
Disease course (years)	0.995	0.002	-2.15	0.032^†^	0.991	1.000
Complication number	1.064	0.012	5.43	< 0.001^‡^	1.040	1.087
HbA1c value	0.976	0.013	-1.84	0.065	0.951	1.002

Note: Estimated by Poisson loglinear function, using generalized estimating equation models; adjusted for clustering on city.

AOR: Adjusted Odds ratio; 95% CI: 95% confidence interval of AOR; SE: Std. Err; †: P < 0.05; ‡: P < 0.01. *: When patients simultaneously had commercial insurance and other plans, they were categorized into the other groups.

## Discussion

This cross-sectional study provided quantitative analysis of the changes in healthcare expenditure and healthcare service utilization caused by the incremental changes of T2DM related complications, medical insurance schemes, individual income and the levels of admission hospitals. To our knowledge, there is no similar data are available for the comparison in China as well as in other developing countries. Although a number of cost-of-illness studies have indicated that T2DM related complications significantly increased the costs for patients [16,19-22], there have been few epidemiological or clinical studies to systematically address the influncing factors of health expenditure of T2DM, especially in developing countries like China.

As expected, there was an association between complications and annual DMC. By using the GEE model, we found that for each increase in number of complications, there was a 33% increase in annual DMC, which quantitatively proved that the high prevalence of diabetic patients who currently live with complications (52% of the outpatients) has significantly increased the economic burden of the type 2 diabetes. Moreover, each increase in the number of complications resulted in extra 6% hospital visits, which may explain the 33% increase of DMC by complications. Therefore, these results strongly indicate that the prevention and treatment of diabetes-related complications could therefore not only improve the life quality and survival of patients but also substantially reduce costs.

Our study show that different insurance schemes were significantly associated with increased annual DMC of T2DM. Although not significant, those covered by urban employee insurance or resident insurance plan had a 24% increase in annual DMC than those without insurance plan; commercial insurance covered patients were found having a significantly higher annual DMC (28% increase). The result implies that the insured patients may overuse the service, as indicated by the widely accepted conventional theory of health insurance based on the concept of moral hazard [23], which is characterized by incremental average cost.

The latest Chinese national health survey in 2003 also revealed that about 73% of people in rural areas who should have sought medical treatment chose not to do so due to cost [24]. However, the results did not correlate the outpatient visit with insurance schemes, which had been reported in other settings [25,26]. It implies that the types of insurance plans might affect the average charges of medical cost per visit, other than frequency of seeking healthcare services for T2DM under the current Chinese Health Insurance System. In fact, the patients covered by health insurance scheme(s) had significantly reduced the proportions of medical cost from pocket, with 19% to 44% decrease in comparison to those uncovered.

China has a urban health insurance system that provides virtually sound coverage for people employed in urban state enterprises and relatively inexpensive coverage for their families. The situation for workers in the rural areas or in urban employment outside the state sector is far more varied. Currently, about 55% of Chinese urban residents are insured through this urban programs[27]. For those who were from rural areas and thus did not hold legal permanent urban residency status might benefit from the Rural Cooperative Medical System. Under this plan, each rural participant pays only 10-20 CNY per year and is then eligible to receive partial coverage of medical expenses, which ranges up to 65 percent of total medical expenses[28]. There were few exceptions for the above two plans, e.g., for unemployed urban residents, who may get insured with a relatively lower reimbursement proportions through a so-called residents insurance plan. When the above plans are absent, patients have to rely on commercial health insurance or individually pay all medical costs.

We found patients with higher income (> 2000 CNY/month) had 23% less payment from their pocket in comparison to those with lower income. Under current China's healthcare system, patients have to pay for treatment out of their own pockets with large differences in quality and access among income groups [29]. Under the current health insurance system in China, the higher income patients are more likely to be covered by insurance or have a better insurance coverage than those with lower income. Our result regarding the OOP is consistent with the known fact. It should be particularly noted that the fact may lead to a potential vicious circle - the poorer the patients are, the relative higher propotion of OOP cost they incur. The result is more worthy of note when it was shown that more than 35% of urban households of China had difficulty to afford healthcare cost, or were poverished by the health costs [30]. The employed outpatients paid less DMC but had to pay more from their pocket (OOP), in comparison to unemployed subjects. It may be caused by a fact that many of the unemployed people were retired residents with high prevalence of chronic diseases but subjected to a lower level of OOP according to the urban insurance plan[31].

In response to growing public concerns over widening inequalities in health, the Chinese Government officially launched another round of the national health system reform plan in early 2009 with a target of 90% health insurance coverage by the end of 2010 and universal coverage of essential healthcare by 2020. [32] Assuming that the reform would be successful, the control of the costs from diabetes may be more likely to depend on the re-distributions of patients in different levels of hospitals or community health centers. In this study, the outpatients admitted at secondary hospitals had 23% more hospital visits, but had 7% less payment of DMC than those seeking medical services at tertiary hospitals. This result is consistent with a study indicating that 6% (997/17148) of tertiary hospitals nationwide consumed 55% of total healthcare expenditure in China [33]. As T2DM can be mostly managed in primary care, our study suggested an urgent need of redistribution patients from tertiary hospitals, charcterized by providing comprehensive and intensive medical services, to facilities where basic healthcare services is easy to be accessed with lower prices.

Furthermore, the association between insulin therapy and higher healthcare costs, that has been reported in other settings(18, 19), was also confirmed in our study. However, we did not find the association between DMC and HbA1c, which had also been reported in other studies [10,34]. The reason may be that cost savings were only significant among a portion of patients who have the highest baseline HbA1c levels (≥10%) but not in patients who have moderate or low HbA1c levels.

The findings from this study may be subject to several limitations. First, the subjects of this study were patients at secondary and tertiary hospitals, which may not be applicable to other groups of patients. The generalizability of the results was also limited by the geographic and demographic characteristics of the study population, considering that only 4 cities were selected as the sites. Second, the cost data of this study, especially for direct non-medical costs and indirect costs, were partially based on patient recall, which may introduce a recall bias. The DMC data that was based primarily on medical history combined with billing information, however, was more precise and accurate. Finally, it was difficult to differentiate T2DM-related complications from coincidental diseases, particularly among the elderly, and possibly some patients were misdiagnosed and therefore stratified incorrectly.

## Conclusion

T2DM related DMC varied with disease characteristics (e.g., the number of complications), whereas the OOP proportion of DMC was largely associated with socioeconomic factors. Our study implied that preventing complications through the use of more effective treatment regimens is important in order to control the healthcare costs of the diseases. With early detection and treatment, it is likely that the serious health consequences of diabetes can be prevented or delayed and thus the costs can be reduced. To reduce the inequity in healthcare expenditures and utilizations of T2DM in different groups, healthcare reform needs to be focused on the reforms of medical insurance system and redistribution of patients in different levels of hospitals.

## Competing interests

This study was sponsored by GlaxoSmithKline (China) Investment Co. Ltd. The authors declare that they has not received fees from the GlaxoSmithKline directly.

## Authors' contributions

WW, BX and CF participated in study design, data collection and interpretation, performed the analyses and drafted the manuscript. JL and HZ participated in the interpretation and drafting of the manuscript. All authors read and approved the final manuscript.

## Pre-publication history

The pre-publication history for this paper can be accessed here:

http://www.biomedcentral.com/1472-6963/10/244/prepub
